# Physicians’ professional autonomy and their organizational identification with their hospital

**DOI:** 10.1186/s12913-018-3582-z

**Published:** 2018-10-12

**Authors:** Domenico Salvatore, Dino Numerato, Giovanni Fattore

**Affiliations:** 1 University “Parthenope” of Naples, Via Generale Parisi 13, 80132 Naples, Italy; 2 Charles University, U Kříže 8/661, Praha 5 - Jinonice, Prague, Czech Republic; 3 Bocconi University, Via Roentgen 1, 20136 Milan, Italy

**Keywords:** Autonomy, Professionalism, Organizational identification, Professional identification, Healthcare, Italy

## Abstract

**Background:**

Managing medical professionals is challenging because professionals tend to adhere to a set of professional norms and enjoy autonomy from supervision. The aim of this paper is to study the interplay of physicians’ professional identity, their organizational identity, and the role of professional autonomy in these processes of social identification.

**Methods:**

We test hypotheses generated according to social identity theory using a survey of physicians working in public hospitals in Italy in 2013.

**Results:**

Higher degrees of organizational and economic professional autonomy are correlated with higher organizational identification. Identification with the profession is positively correlated with identification with the organization.

**Conclusions:**

Although the generalizability of our results is limited, this study suggests that organizations should support the organizational and economic autonomy of their physicians to project an organizational identity that preserves the continuity of a doctor’s self-concept and that is evaluated as positive by doctors. As a result, organizations will be able to foster organizational identification, which is potentially capable of inducing pro-social organizational behavior.

**Electronic supplementary material:**

The online version of this article (10.1186/s12913-018-3582-z) contains supplementary material, which is available to authorized users.

## Background

Healthcare organizations typically employ many professionals, workers who are deemed to adhere to a set of professional norms [1] and to enjoy autonomy from supervision [2]. Because of the nature of these professionals’ work, most management scholars maintain, following Mintzberg’s perspective [3], that professionals should enjoy a high level of autonomy from supervision when using their professional skills and knowledge; control of their behavior mainly occurs by social and self-control mechanisms [4]. Hence, at first sight, medical autonomy might seem to contradict the notion of centralized organizational authority [5]. However, recent studies suggest that it may be time to move away from this binary approach and acknowledge a more nuanced perspective [6].

How can organizations foster social and self-control mechanisms that induce professionals to consider their organization’s interests while working with a high degree of autonomy? In this paper, we will focus on one possible approach answering to this question, namely, organizational identification. We will, therefore, explore the interplay of physicians’ professional identity, their organizational identity, and the role of professional autonomy in these processes of social identification in the highly professionalized context of hospital care.

In the context of increasing organizational responsibilities of healthcare professionals, the logics of professionalism and managerialism are not necessarily treated as opposing and mutually exclusive [7, 8]. These developments contributed to the frequent emergence of hybrid professional-management roles (e.g. [9–11]). In this paper, we study this link through the lens of social identity theory [12, 13] and by employing the construct of social identification. Because of its focus on the influence of group membership on individual behavior, social identity theory enables the development of new insights into the behavior of professionals in organizations.

We use social identity theory to develop hypotheses regarding the relationships among perceived autonomy, identification with the profession, and identification with the organization; then, using 220 responses to a survey of physicians working in public hospitals in Italy, we empirically test these hypotheses. The objective of this study is twofold: first, to analyze the relationship between organizational identification related to different dimensions of professional autonomy, and second, to examine the relationship between organizational and professional identification.

## Professional autonomy of physicians

Professional autonomy is the legitimate control that the members of an occupation exercise over the organization and the terms of their work [14]. Individual-level studies of physicians’ autonomy inside organizations have examined professional autonomy in different domains [15, 16]. Following these and other studies, we identified three main dimensions of autonomy: 1) clinical work freedom, 2) social and economic work freedom, and 3) influence on organizational decisions.

*Clinical work freedom* is the most obvious type of professional autonomy; it refers to the ability of doctors to decide to provide care to a patient without being limited by organizational procedures, financial concerns, performance measurement systems, or managerial control. Studies analyzing this type of autonomy include, for example, physicians’ control over decisions regarding which tests and examinations to order, which drugs and procedures to prescribe, and to whom referrals should be made [17–19]. Restrictions on such autonomy often define aggregated measures such as pre-set budgets, identify outliers with atypical treatment patterns, or channel certain treatments through a gatekeeper [20].

*Social and economic work freedoms,* such as control over earnings and control over the nature and volume of tasks, exist independently of but in relation to clinical work freedoms [21]. Control over the nature and volume of medical tasks represents doctors’ ability not to be managed in the industrial sense but rather to determine their own movements, priorities, schedules and workloads [20]. We argue that social and economic work freedoms, as a component of professional autonomy, function universally across different healthcare systems, but the effect of healthcare systems on specific social and economic freedoms can differ. The institutional arrangements of a healthcare system greatly influence this dimension of autonomy: the social and economic freedom available to a state-employed physician in a country with universal healthcare will likely be different from the social and economic freedom available to a self-employed physician in a country with voluntary health insurance.

*Influence on organizational decisions* refers to doctors’ voices in organizational and managerial choices and their ability to influence the manner in which their unit and their hospital function. Power to influence organizational and sub-unit decisions depends on the division of labor, namely, on the control of strategic uncertainty [22]. Professionals tend to incorporate organizational issues into their professional domains [23], and higher skill levels may be correlated with higher participation of professionals in decision-making [24].

## Organizational and professional social identification

Social identification is the perception of oneness with or the sense of belonging to a group [25]; individuals define themselves in terms of their group membership and ascribe characteristics that are typical of the group to the self [26]. Social identification is ‘that part of an individual’s self-concept which derives from his knowledge of his membership of a group (or groups) together with the value and the emotional significance attached to the membership’ [27]. Social identification influences how the self is defined by group membership and therefore influences individual behavior.

One type of social identification is organizational identification, which is the extent to which an individual’s self-concept contains attributes identical to those of the perceived organizational identity. The strength of an individual’s organizational identification depends on how well the image of an organization preserves the continuity of the individual’s self-concept, provides distinctiveness, and enhances self-esteem [28]. Organizational identification is correlated with a wide range of work-related positive attitudes and behaviors such as commitment, organizational citizenship behaviors, and job turnover [29–31].

An individual can identify with multiple groups and have a job-related identity in addition to identities based on gender, age, ethnicity, or nationality. Individuals may also have multiple job-related identities; for instance, they can identify with both their organization and their profession [32, 33].

Given the sociological tradition of treating professionalism and managerialism as opposing concepts and given that the word *organization* is sometimes misleadingly used as a synonym for *bureaucracy,* one might expect organizational identification and professional identification to be inversely correlated. On the contrary, direct correlations reported in the few published studies that have measured both identifications suggest that organizational and professional identification are positively correlated [32–34].

The images of one’s organization and of one’s profession are subjectively perceived by each individual as is the degree of identification with these two groups. However, because being part of an organization does not imply a loss of autonomy, the two identities do not ontologically exclude one another. Organizations coordinate not only by rules and supervision but also by delegating to and empowering their employees; being part of an organization may offer opportunities that an individual does not have.

The idea that organizational identification and professional identification are not inversely correlated is consistent with recent scholarship in healthcare management and governance that has demonstrated that the roles of managers, who represent organizations, and physicians, who represent professional communities, have become blurred and hybrid (e.g. [11]). At the same time, these processes of hybridization do not automatically contribute to an increased involvement of doctors in decision-making processes [10].

## Hypotheses

Viewing professional autonomy and organizational control through the lens of social identity theory, we are interested in two research questions. First, is perceived professional autonomy, particularly its three dimensions (clinical, economic, and organizational), related to organizational identity? Second, what is the relationship between professional and organizational identities?

Today, professional image is greatly influenced by decades of discourse on the professional power, autonomy, and self-regulation held by professional elites, scientific societies, physicians’ unions, and sociologists. Because autonomy is such an important attribute of professional groups [15], we hypothesize that individual physicians who perceive that their organization allows them considerable autonomy will perceive an organizational image that preserves the continuity of their self-concept and enhances their self-esteem, thus creating a strong organizational identification. Therefore, considering the three dimensions of professional autonomy outlined above, we hypothesize the following:

- *H1a*: Clinical work freedom is positively related to organizational identification.
- *H1b*: Social and economic work freedom is positively related to organizational identification.
- *H1c*: Influence on organizational decisions is positively related to organizational identification.

We also hypothesize that physicians holding managerial responsibilities (e.g. clinical directors and other hybrid doctor-manager positions) identify with the organization more strongly than other physicians because organizations give them more power in organizational decision-making, more complex responsibilities, and greater non-clinical autonomy. A doctor-manager is entitled with more power given her organizational role. Thus, increased breadth of responsibilities is directly linked to job autonomy [35]. Therefore, we hypothesize the following:

- *H2:* The effect of managerial responsibilities on organizational identification is mediated by *social and economic work freedoms* and *influence on organizational decisions*.

**Fig. 1:**
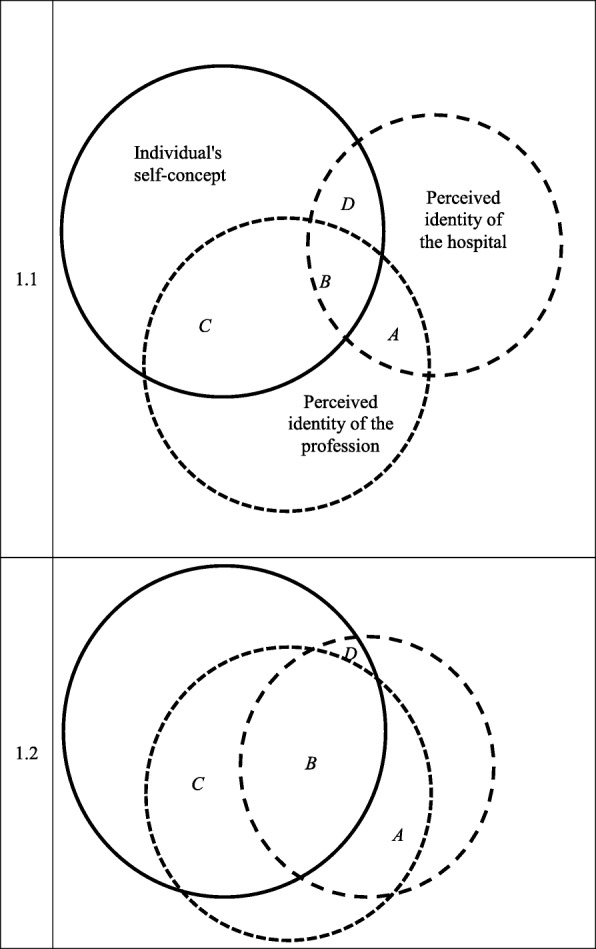
Identity overlaps

If identification is the extent to which an individual’s self-concept contains attributes identical to those of the perceived collective identity, then professional and organizational identification will be positively correlated when their respective images are characterized by a set of overlapping attributes or when the attributes of one are the complement of the other. This idea may be more clearly explained by the graphical representation in Fig. 1: Each individual has an image of herself and perceives an image of her organization and an image of her profession. Organizational identification, which is the extent to which an individual’s self-concept contains attributes identical to those of the perceived organizational identity, could be represented as the sum of the area marked D plus the area marked B in Fig. 1. Similarly, professional identification could be represented as the sum of areas C and B. In Fig. 1.2, the circles representing ‘Perceived identity of the hospital’ and ‘Perceived identity of the profession’ overlap more than in Fig. 1.1, resulting in a larger B area. The larger this overlap (represented by the B area) is, the higher the correlation is between organizational and professional identification.

According to Rousseau [36], ‘Identification occurs when an individual and an organization have common interests that dominate their differences and the individual perceives that his or her relationship to the organization forms an “us” (e.g. among individual members of an ad hoc taskforce working together to meet a deadline).’ The medical profession and a hospital are two institutions with multiple and complex goals; however, many of these goals are shared by the two institutions. Therefore, we hypothesize the following:

- *H3:* Professional identification is positively related to organizational identification.

Both the professional and organizational identities reside within the job identity of a doctor. The correlation between professional identity and organizational identity is, therefore, the extent to which the individual’s self-concept overlaps with their job identities.

## Methods

To answer the research questions and test the hypotheses, we conducted a survey among doctors from all the public hospitals in two regions in northern Italy (Lombardy and Emilia-Romagna). All the hospitals in our survey are owned by their regional government and mainly provide care which is financed by the Italian NHS. The doctors working for these hospitals are salaried employees of their hospital.

In Italy, public hospitals are mandated to publish the contact information of their doctors. Using the websites of each hospital, we identified the name, the email address and the physical office address of each cardiologist and orthopedist employed by the hospital. In 2013, we first sent a printed questionnaire to those doctors thus identified with a pre-stamped envelope for returning the questionnaire. In the subsequent month, we sent two reminder emails with a link to a web version of the same questionnaire via traditional mail. In the cover letter, respondents were guaranteed anonymity and were assured that hospital management was not involved in this study.

Of the 1703 questionnaires sent, 220 were returned, yielding a 13% response rate; one of the 220 questionnaire was empty and thus it has not been usable for further analysis, for which the final sample is equal to 219. The relatively low response rate is most likely because of the manner in which the survey was administered to a busy population: medical doctors are targeted by many studies. Originally we run models only with observed data, but later we have input missing values for the scales that were based on more than one question: in particular we input values for the three autonomy scales (clinical work freedoms, influence on organizational decisions, social and economic work freedoms) and missing values have been replaced with the mean of the other values of the same scale. We did not observe any statistically significant difference in the response rates based on the known variables for the entire population (gender, medical specialty, and region). Table 1 contains demographic information of the sample.

**Table 1:** Sample characteristics

	Emilia Romagna	Lombardy	Total
Number of respondents (%)	85 (39%)	134 (61%)	219
Gender			
Female (%)	17 (21.5%)	32 (25%)	49 (23.7%)
Male (%)	62 (78.5%)	96 (75%)	158 (76.3%)
Age (SD)	49 (8.9)	51 (8.2)	50.2 (8.6)
Medical Specialty			
Cardiology	49 (42.3%)	57 (42.5%)	126 (42.5%)
Orthopedics	36 (57.7%)	77 (57.5%)	93 (57.5%)
With managerial responsibilities			
Yes	23 (28%)	39 (31%)	62 (29.8%)
No	59 (72%)	87 (69%)	146 (70.2%)
Publishing Research			
Yes	48 (58.5%)	88 (68.8%)	136 (64.8%)
No	34 (41.5%)	40 (31.2%)	74 (35.2%)

Individual-level studies of physicians’ autonomy inside organizations have examined professional autonomy in different domains [15, 16]. By reviewing these studies, we identified three main dimensions of autonomy: (i) clinical work freedom, (ii) social and economic work freedom, and (iii) influence on organizational decisions.

Then, we identified the scales that were employed in previous empirical studies, critically assessed them and selected the items that suited the measurement needs of our survey. These items were translated into Italian and then back-translated into English by two different researchers to check for consistency in meaning; the items were subsequently pretested using three doctors of differing specialties.

The scales are reported in Additional file 1.

### Dependent variable

*Organizational identification* was measured using Bergami & Bagozzi’s [37] graphic scale that conceptualizes identification in terms of the cognitive distance or space between an individual and a collective. This scale focuses mainly on the cognitive dimension of the construct. The advantage of this graphic scale compared to a multi-item verbal scale is that interrupting respondents’ response style [38] reduces the bias caused by common methods variance. The response range for this scale has been from 1 to 8.

### Independent variables

The Clinical Work Freedoms Scale, the Social and Economic Work Freedoms Scale and the Influence on Organizational Decisions Scale were measured using a seven-point Likert scale (− 3 = strongly disagree to + 3 = strongly agree).

*Clinical work freedoms* were operationalized using items by Baker & Cantor [19] and from Kilic, Arslan, Leblebici, Aydin, & Oktem [39]. An example of the items on this scale is as follows: ‘I can hospitalize any patient who, in my opinion, requires it.’ The Cronbach’s alpha reliability coefficient for this scale was 0.83.

*Social and economic work freedoms* were operationalized according to the features of this autonomy dimension [21]. The Cronbach’s alpha reliability coefficient for this scale was 0.87.

*Influence on organizational decisions* was operationalized using items from Kilic et al. [39]. An example of items on this scale is as follows: ‘I have influence on managerial practices in my unit.’ The Cronbach’s alpha reliability coefficient for this scale was 0.75.

*Managerial responsibilities* were measured using a dichotomous variable coded ‘1’ if the respondent declared having a formal managerial role.

*Professional identification* was operationalized as the perception of oneness with the professionals in the same medical specialty (either cardiology or orthopedics). Professional identification was measured by using the identical graphic scale used for organizational identification and substituting the word ‘hospital’ for the medical specialty of the respondent. In this scale, as in the organizational identification scale from which it is drawn, the response range has been from 1 to 8.

### Control variables

In our analyses, we controlled for the following variables, which may also affect organizational identification: the region in which the hospital is located, the medical specialty of the physician, her gender, and involvement in research measured through declared publications.

## Results

Table 2 reports the means, standard deviations and correlations of the above-mentioned variables. Table 3 shows the results of linear regressions with organizational identification as the dependent variable.

**Table 2:** Means, standard deviations and correlations

	n	Mean (SD)	1	2	3	4	5	6	7	8	9
1.Organizational Identity (1–8)	209	41.7 (1.93)									
2. Region (Emilia Romagna =1)	219	0.61 (0.49)	0.09 (0.18)								
3. Medical Specialty (Cardiology = 1)	219	0.42 (0.5)	−0.01 (0.85)	− 0.00 (0.97)							
4. Gender (female =1)	207	0.24 (0.43)	−0.1 (0.12)	− 0.03 (0.70)	0.31 (0.00) **						
5. Publishing research (1 = yes)	210	0.65 (0.48)	0.16 (0.02) *	−0.10 (0.13)	0.16 (0.02) *	−0.09 (0.2)					
6. Managerial responsibilities (1 = yes)	208	0.3 (0.46)	0.26 (0.00) **	−0.03 (0.66)	0.01 (0.90)	0.22 (0.00) **	0.27 (0.00) **				
7. Clinical work freedom (− 1 to 1)	218	0.83 (1.57)	0.28 (0.00) **	−0.04 (0.56)	0.12 (0.06)	0.13 (0.06)	0.09 (0.03)	0.15 (0.03) *			
8. Social and Economic Work Freedom (− 1 to 1)	217	−0.65 (1.63)	0.38 (0.00) **	−0.16 (0.01) *	−0.03 (0.57)	− 0.12 (0.08)	0.21 (0.00) **	0.34 (0.00) **	0.26 (0.00) **		
9. Influence on Organizational Decisions (− 1 to 1)	218	−0.27 (1.47)	0.42 (0.00) **	−0.04 (0.52)	0.02 (0.76)	−0.06 (0.35)	0.14 (0.04)	0.34 (0.00) **	0.41 (0.00) **	0.52 (0.00)	
10. Professional Identity (1–8)	202	5.87 (1.69)	0.15 (0.04)	0.02 (0.78)	−0.02 (0.77)	0.25 (0.00) **	−0.07 (0.32)	−0.01 (0.89)	0.18 (0.01) *	0.02 (0.81)	0.16 (0.02)

*p* < 0.05; ** *p* < 0.01

**Table 3:** Determinants of organizational identification (ordinary least square)

	Dependent Variable: Organizational Identity (scale 1–8);						
	B Coefficients and 95% Confidence Intervals						
Age	0.03 (0.03; 0.07)*	0.00 (−0.03; 0.03)	−0.01 (− 0.04–0.02)	−0.00 (− 0.03; 0.03)	−0.01 (− 0.04;0.02)	0.00 (− 0.03; 0.04)	−0.01 (− 0.04; 0.02)
Region (Emilia Romagna =1)	0.48 (0.03–1.07)*	0.60 (0.09; 1.12) *	0.78 (0.27–1.3) *	0.62 (0.12; 1.11)*	0.74 (0.26–1.2)*	0.56 (0.4–1.08)*	0.75 (0.25–1.24)*
Medical Specialty (Card. = 1)	−0.22 (− 0.84; 0.25)	−0.33 (− 0.87–0.20)	−0.24 (− 0.76–0.28)	−0.29 (− 0.80;0.23)	−0.30 (− 0.80; 0.21)	−0.43 (− 0.98–1.3)	−0.41 (− 0.93–1.1)
Gender (female =1)	−0.33 (− 0.98; 0.32)	−0.47 (− 1.12; 0.18)	−0.27 (− 0.89–.035)	−0.29 (− 0.90;0.32)	−0.32 (− 0.93; 0.29)	−0.44 (− 1.12;0.23)	−0.43 (− 1.07;0.21)
Publishing research (1 = yes)	0.30 (− 0.31; 0.92)	0.33 (− 0.23; 0.91)	0.22 (− 0.34–0.78)	0.27 (− 0.27;0.82)	0.20 (− 0.34;0.74)	0.56 (− 0.19: 1.14)	0.33 (− 0.21: 0.88)
Managerial responsibilities (1 = yes)	.750 (0.12; 1.37) **	0.63 (− 0.00; 1.28)	0.42 (− 0.21; 1.05)	0.37 (− 0.25–1.00)	0.24 (−0.39; 0.85)	0.77 (0.13; 1.41)*	0.31 (−0.31; 0.94)
Clinical work freedom (− 1 to 1)		0.25 (0.09; 0.42)*			0.11 (−0.06; 0.27)		0.13 (−0.03; 0.30)
Social and Economic Work Freedom (− 1 to 1)			0.41 (0.24–0.58) **		0.27 (0.09; 0.45)**		0.27 (0.10; 0.45) **
Influence on Organizational Decisions (− 1 to 1)				0.44 (0.27–0.612)**	0.28 (0.08; 0.48)**		0.21 (0.14;0.42) *
Professional Identity (− 1 to 1))						0.20 (0.04; 0.36) *	0.15 (−0.01;0.3)
Intercept	3.5 (1.7; 5.4)*	3.6 (1.8–5.4)*	3.1 (1.1; 5.05)*	4.1 (2.4; 5.8)*	4.5 (2.8–6.2)*	2.26 (0.23–4.28)*	3.57 (1.64–5.5)*
Adjusted R2	0.05	0.12	0.17	0.18	0.22	0.12	0.24
F statistics	F(5, 193) = 3.47; *p* = 0.007	F(6, 191) = 6.19; *p* < 0.001	(F6, 190)==8,21; *p* < 0.001	(F6, 190)==8,65; *p* < 0.001)	(F8, 188)==9,42; *p* < 0.001)	(F8, 185)==5,47; *p* < 0.001)	(F9, 182)==9,23; *p* < 0.001)
n	199	198	197	197	197	192	192

*p* < 0.05; ** *p* < 0.01

Hypotheses 1a, 1b, and 1c posited a positive influence of the three dimensions of autonomy on organizational identification. As Table 3 shows, we did not observe a significant effect of clinical work freedoms on organizational identification, although we observed positive effects of the two non-clinical dimensions of autonomy: social and economic work freedom and influence on organizational decisions. This result may suggest that physicians do not perceive their clinical freedom as being relevant to their organizational identity, whereas they perceive their freedom to organize their own work and their participation in organizational decision making as being relevant to their organizational identity.

Hypothesis 2 suggested that greater social and economic work freedoms and influence on organizational decisions induce doctors with managerial roles to identify more closely with their organizations. Table 3 shows that the effect of managerial responsibilities on organizational identification is positive and significant but that it becomes non-significant once autonomy dimensions and professional identification are added to the regression model.

**Fig. 2:**
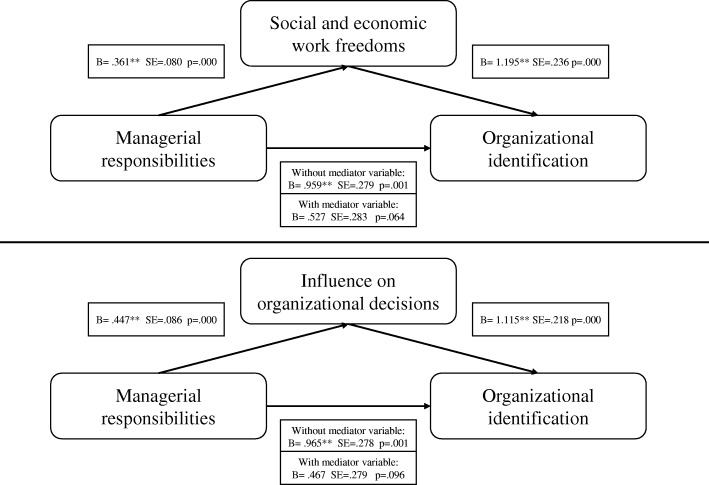
Graphical representation of the results of the mediation analysis (H2)

To test the mediation relationships, we followed Sobel’s approach [40]. Results show that the R–squared value of the model with *Social and economic work freedom* as mediation variable is 0.14 (F(2, 168) = 16.7; *p* < 0.001). Sobel test supports the mediation hypothesis (coeff = 0.53, SD = 0.128, Z = 0.337, *p* < 0.001). *Managerial responsibilities*, without the mediator effect of *social and economic work freedom*, shows a significant relation with *organizational identification* (B = 0.959, SE = 0.28); however, when this mediating variable has been entered into the model (relation between *managerial responsibilities* and *social and economic work freedom*: B = 0.361, SE = 0.07; relation between *social and economic work freedom* and *organizational identification*: B = 1.195, SE = 0.14), *managerial responsibilities* showed no significant relationships with *organizational identification* (B = 0.527, SE = 0.28). The same goes for the mediating variable of *influence on organizational decisions (*R^2^ = 0.17; F(2,199) = 20.4; *p* < 0.001): *managerial responsibilities*, without the mediator effect of *influence on organizational decisions*, shows a significant relation with *organizational identification* (B = 0.965, SD = 0.278); however, when this mediating variable has been entered into the model (relation between *managerial responsibilities* and *influence on organizational decisions*: B = 0.447, *p* = 0.086); relation between *influence on organizational decisions* and *organizational identification*: B = 1.115, *p* = 0.215), *managerial responsibilities* showed no significant relationships with *organizational identification* (B = 0.467, SD = 0.279). The *influence on organizational decisions* takes the role of mediator in this relationship and then confirms completely the Hypothesis 2. Figure 1 graphically represents the results of this same mediation relationship and used a Sobel [41] test (coeff = 0.49; SD = 0.136; Z = 3.669, *p* = 0.002). As shown in Fig. 2, the results support the mediation hypotheses.

The regression results reported in Table 3 also support Hypothesis 3, showing a positive relationship between professional identification and organizational identification. As mentioned in the hypotheses section, we do not hypothesize a direct causal effect of professional identification on organizational identification; however, we do believe that the positive association between these two social identifications is caused by the overlapping goals of the two social groups.

## Discussion

In this article, we explore how physicians’ identification with their hospital is related to the type and degree of autonomy they experience in their work, their managerial responsibilities, and their identification with their profession. Using data from a survey of specialists working in Italian public sector hospitals, we observed that doctors identify more with their hospitals if they experience freedom in the organization of their own work and if they perceive that they have the power to influence organizational decision making. We did not observe any significant effect of the clinical freedom experienced by physicians on their identification with their hospital. We also observed that doctor-managers tend to identify more with their hospitals when they enjoy more freedom in the organization of their own work and when they have the power to influence their units and their hospital choices. Finally, we observed that hospital identification and professional identification do not necessarily conflict but may be positively correlated.

Social identification processes provide a sound explanation of how the macro-level phenomena of professionalism and professionalization described by a consolidated sociological tradition [1, 41–43] influence and are influenced by the behavior of the individual professional. Individual professionals are shaped by and shape, produce, and reproduce the norms and scripts of their profession [44]. This theoretical link between the collective level of the profession and the individual level of the professional is, in our opinion, a great advantage to studying highly professionalized work contexts.

The idea that each individual has multiple identifications [32, 33, 45–47] accurately describes the fact that individuals appear to have similar attitudes toward their profession and the organization in which they practice their profession. Social identity theory, therefore, allows insights beyond those of the hegemony/resistance framework [7] that is implicit in many studies on the effect of management on professionalism in organizations. As mentioned above, we do not believe that there is a direct causal relationship underlying the positive correlation between professional identification and organizational identification; however, we do believe that some portions of the professional and organizational identity perceived by physicians working in hospitals may overlap. One possible explanation for our findings is that the concepts of organizational freedom and organizational power are currently components of the general perception of professionalism. Future studies should investigate the content of the identities of medical professions and of hospitals (these identities are also context-specific).

Physicians’ identification with their hospital is a relevant dependent variable in healthcare management. Many activities in healthcare are complex, and classical bureaucratic control based on hierarchical supervision and standardization of procedures is often not feasible [3, 4]: managerial intervention in employees’ self-constructions is a control mechanism. Alvesson & Willmott [48] defined this control mechanism as ‘identity regulation’, and although this mechanism is not always intentional and effective, it deserves further investigation in healthcare settings.

## Conclusions

The main practical implication of this study is in providing initial evidence that organizations should cede autonomy to professional employees to project an organizational identity that preserves the continuity of the doctor’s self-concept and that is evaluated as positive by doctors. By doing so, organizations can foster organizational identification, which is a key psychological state reflecting the underlying bond that exists between the employee and the organization. This manner of regulating physicians’ identity may be important in healthcare settings in which alternative control mechanisms are weaker than in other work settings.

Further studies should focus on a more detailed understanding of the relationship between clinical autonomy and organizational identification. The missing relationship between these two variables may be caused by other variables that are beyond the scope of our analysis. More specifically, clinical work freedom is related not only to organizational context—as in the cases of social and economic freedom and influence over organizational decisions—but also to the broader logic of contemporary healthcare management, which includes the promotion of an evidence-based medicine culture, clinical guidelines, and national regulations. Further examination of how organizations in different contexts are intertwined with regulation mechanisms outside hospitals would provide a more detailed understanding of clinical autonomy in the organizational context.

Our empirical investigation of these phenomena is limited by the study design, which used a single-source, cross-sectional survey. Our study considers the processes of social identification in two Italian regions, by analyzing two groups of practitioners, namely the cardiologists and orthopedic surgeons. Therefore, our results may be influenced by the context in which we investigated the phenomenon; the congruence of organizational and professional identity may be different in public hospitals in Europe than in for-profit hospitals in other cultures.

Another limitation could be in the identity measure we used. Although being previously validated, the identity measure focuses on the cognitive dimension of identity (rather than the affective dimension) [49, 50]. Furthermore, the use of the same type of scale to measure organizational and professional identification risks create a common method bias.

Moreover, due to the relatively low response rate of 13%, the results of this preliminary study cannot be generalized. Nevertheless, this study is still among the few quantitative studies of professionalism inside organizations and among the few examining this issue using social identity theory. Despite these limitations, indeed, this study contributes to add empirical evidence to the scholarship focusing on the relationship among management and professionalism at the individual level of analysis using organizational behavior’s constructs, guaranteeing a new perspective of analysis to a fundamental issue in health services research. This new theoretical and empirical approach is useful in encouraging the re-opening of an important discussion: how professionalism influences individual behavior inside organizations which is a central issue in healthcare management.

## Additional file

Additional file 1: Autonomy scale (a seven-point Likert scale (-3 = strongly disagree to +3 = strongly agree)). (DOCX 58 kb)
