# Supplementary material for: Physicians’ professional autonomy and their organizational identification with their hospital
Source: BMC Health Serv Res. 2018 Oct 12;18:775. doi: 10.1186/s12913-018-3582-z (PMC6186093; doi:10.1186/s12913-018-3582-z)
Supplement: Supplementary file 1 — Autonomy scale (a seven-point Likert scale (-3 = strongly disagree to +3 = strongly agree)). (DOCX 58 kb) [file 12913_2018_3582_MOESM1_ESM.docx]

Additional file 1: Autonomy scale (a seven-point Likert scale (-3 = strongly disagree to +3 = strongly agree)).

**Clinical work freedoms**

- I can hospitalize any patient who, in my opinion, requires it.
- I can choose medically required diagnostic tests without any restrictions.
- I can prescribe any drug that is beneficial to the patient.

**Influence on organizational decisions**

- I have influence over managerial practices in my unit.
- I have influence over managerial practices in my hospital.
- My opinion is important in clinical audit processes.

**Social and economic work freedoms**

- I am able to choose the nature of the work I perform.
- I can choose my workload.
- I am free to plan my work myself.
- I can choose my work schedule.
